# Surveillance data and progress assessment of the targets of the WHO Global Leprosy Strategy 2016–2020: A retrospective registry-based analysis in Ghana

**DOI:** 10.1371/journal.pntd.0014604

**Published:** 2026-08-10

**Authors:** Anna Katharina Njillo, Stephen Adom Boateng, Till Omansen, Ruth Ansah, Johannes Mischlinger, Michael Ramharter, Benedict Okoe Quao, Mirjam Groger

**Affiliations:** 1 Center of Tropical Medicine, Bernhard Nocht Institute for Tropical Medicine & I. Department of Medicine University Medical Center Hamburg-Eppendorf, Hamburg, Germany; 2 National Leprosy Control Programme, Ghana Health Service, Accra, Ghana; 3 Department of Virology, Bernhard Nocht Institute for Tropical Medicine, Hamburg, Germany; 4 German Center for Infection Research (DZIF), Partner Site Hamburg–Lübeck–Borstel–Riems, Germany; 5 Centre de Recherches Médicales de Lambaréné (CERMEL), Lambaréné, Gabon; University of Bremen: Universitat Bremen, GERMANY

## Abstract

**Background:**

The WHO Global Leprosy Strategy 2016–2020 aimed at early detection of new leprosy cases, reduction of grade-2 disabilities (G2D), and stopping any discrimination of people afflicted with leprosy while pointing out the importance of leprosy control programmes. In this retrospective study, data obtained from the Ghanaian National Leprosy Control Programme (NLCP) prior to the Covid-19 pandemic were analysed and compared with the key targets of the Global Leprosy Strategy 2016–2020.

**Methodology/principal findings:**

Demographic as well as clinical data from the registry of the Ghanaian NLCP from 2017 to 2019 were reviewed and described. Evaluated targets and indicators for WHO programme monitoring included new case detection rates, proportion of new cases with G2D, biological sex, paediatric cases, proportions of multibacillary (MB) and paucibacillary (PB) cases and treatment completion rates. Data were further categorized according to region and year. A total of 816 new leprosy cases were evaluated in this analysis. Of these, 358 (43.9%) were female cases and 33 (4. 0%) were paediatric cases. While the annual new case detection rate per 100,000 population in Ghana was 0.86 in 2017, 0.93 in 2018, and 0.90 in 2019, individual regions, particularly the Upper West Region, remained consistently above the threshold of 1 new case per 100,000 (4.32, 4.71, 6.12). Of all new cases, 7.6% (n = 62) presented with G2D, the rate remained <1 per million population in each of the three years. One paediatric G2D case was reported in 2019. Annual treatment completion rates of MB and PB cases were well over 90% in 2017 and 2018.

**Conclusions/significance:**

The NLCP made partial progress towards the WHO targets. While the analysis demonstrates that Ghana was on track regarding the elimination of leprosy, the presence of one paediatric case with G2D at diagnosis highlights the need for continued targeted interventions and enhanced surveillance to achieve elimination goals.

## Author summary

Although leprosy is not being considered as a worldwide public health problem since 2000, it remains a public health challenge in a multitude of countries, including on the African continent. The World Health Organization (WHO) aims to eliminate leprosy worldwide and accompanies this process by strategic guidelines. Data collected via the National Leprosy Control Programme in Ghana between 2017–2019, reflecting the time before the Covid-19 pandemic, were analysed and compared to the objectives defined in the WHO Global Leprosy Strategy 2016–2020. Our results demonstrate a consistently low number of new leprosy cases and good treatment completion rates in Ghana. However, the proportion of patients diagnosed with debilitating disability remains high, indicating delays in diagnosis and potential gaps in community awareness or access to care. Regional disparities in disease burden were also observed. Our findings suggest that further efforts must be made in leprosy surveillance in general and improved monitoring of the disease in areas with a high disease burden should be established. A continued investment into the health system as well as efforts in reducing the stigma associated with leprosy could further aid early diagnosis and help Ghana to reach elimination targets in the post-Covid era.

## Introduction

Leprosy is a chronic infectious disease mainly caused by *Mycobacterium leprae*. Transmission of the infectious agent most likely occurs by droplet infection via the mouth and nose and requires prolonged close contact with an infectious person [1]. The incubation period of leprosy is long and variable and can be up to 20 years [2]. Affected children seem to be more likely to transmit the infection to other household members than affected adults [3]. The clinical diagnosis of leprosy is based on characteristic skin lesions and enlargement of peripheral nerves [1]. In addition, bacilli may be detected microscopically in slit-skin smears (SSS) [4]. Leprosy is classified according to the number of lesions as paucibacillary (PB; 1–5 skin lesions) or multibacillary (MB; > 5 skin lesions or presence of bacilli in SSS). The currently recommended multidrug therapy (MDT) includes rifampicin, dapsone and clofazimine for 6 months for patients infected with PB leprosy and 12 months for patients infected with MB leprosy [5]. An effective control of leprosy can be achieved by early detection of the disease followed by early treatment with MDT [6].

Leprosy belongs to the Neglected Tropical Diseases (NTDs) [1]. While the countries with the highest leprosy case numbers are located in South America and Asia, African countries have made part of the ten countries worldwide with the highest numbers of new case detection for many years as well [7,8]. Although leprosy was declared eliminated in the year 2000, an estimate of 208,613 new cases were still diagnosed worldwide in 2019 with an estimate of 20,184 of them originating on the African continent [9]. According to the latest WHO estimates, this situation persists as 19,171 new leprosy cases were reported in Africa in 2024. [10]. The World Health Organisation’s (WHO’s) ultimate goal is zero cases, and this process is guided by a written strategy. The WHO’s Global Leprosy Strategy 2016–2020 defined three main goals for the elimination of leprosy: reduction of cases with grade 2 disability (G2D) among paediatric leprosy patients to zero, reduction of new leprosy cases with G2D to less than one case per million population and reduction of countries with legislation allowing leprosy-associated discrimination to zero [11]. In addition, other so-called ”programme performance indicators” were defined to enable monitoring of progress [11,12]. Another aim of WHO’s Global Leprosy Strategy 2016–2020 was to further eliminate leprosy at the subnational level by initiating action, ensuring accountability and promoting inclusivity, to set the course for reaching sustainable development goal 3 good health and wellbeing for all by 2030 [12].

Ghana is a West African country with an estimated population of about 32 million in 2020 [8,9,13]. This manuscript presents data from the National Leprosy Control Programme (NLCP). MDT was introduced in the country in 1984, and by 1991, MDT was available to 100% of the diagnosed patient population. Although Ghana is not among the leading countries regarding leprosy incidence and prevalence, the NLCP puts extensive efforts in the improvement of leprosy management and control in the country. Since 2000, the number of new cases in Ghana has been reduced by 80%. Since 2014, the NLCP in Ghana has routinely collected leprosy surveillance data [14]. While country-specific data are also reported to and evaluated by the WHO on a regular basis, different regions of Ghana face individual challenges related to leprosy. To be able to address and monitor these challenges in a more targeted way, this retrospective registry-based cross-sectional analysis aimed to evaluate the available core programme indicators and elimination goals of NLCP in relation to the WHO’s Global Leprosy Strategy 2016–2020. To allow for more detailed monitoring and more targeted public health interventions by the NLCP, this descriptive analysis was done not only at the national but also at the regional level.

## Methods

### Ethics statement

For this study the ethical approval for was obtained from the Ethics Committee of the Ghana Health Service (Reg.-Nr. GHS-ERC 052/03/21). In this retrospective analysis, anonymized data were used.

The NLCP routinely collects leprosy-related data from health care facilities all over Ghana in a country-wide registry. Until 2020, surveillance data were reported monthly by health centres to the region and then to the NLCP. Based on these data, the NLCP went back to the health centres to request more details and compiled a line list, which served as basis for this analysis. All patients who were newly diagnosed with leprosy between 2017 and 2019 were included in this retrospective cross-sectional analysis of data from the NLCP-related registry. Patients with first leprosy detection in 2019 were, however, excluded from the calculation of treatment completion rates as for them treatment completion was to be reached only beyond the observational period. Relapse cases and cases that newly presented in 2016 (n = 4), were excluded as well as cases with discrepant information between age and year of birth (n = 2). Another case was excluded due to discrepant information between treatment date and year (n = 1). No age restrictions were applied. In 2019, six new regions and additional districts were established in Ghana [15]. This descriptive data analysis considers the 10 former regions which are Ashanti, Brong Ahafo, Central, Eastern, Greater-Accra, Northern, Upper East, Upper West, Volta, and the Western Region.

Ethical approval for this analysis was received by the Ethics Committee of the Ghana Health Service (Registration number: GHS-ERC 052/03/21). Unique identifiers were allocated by NLCP staff before the dataset was shared for further cleaning and analysis. Variables extracted from the database were year of first detection, gender, year of birth/age, residence, disease classification, skin smear date and result, maximum disability grade at diagnosis, date of treatment initiation, and treatment attendance. In order to compare the data available from the registry with the performance milestones defined in Global Leprosy Strategy 2016–2020 the following core programmatic indicators were calculated: annual new case detection (total number of new leprosy cases and rate per 100,000 of the population), proportion of grade 2 disability (G2D) cases among new cases, proportion of paediatric cases among new cases, proportion of MB cases among new cases, proportion of female cases among new cases, treatment completion rate of MB cases, and treatment completion rate of PB cases. Treatment was regarded as completed if a MB patient came to collect the medication 12 times within the first 18 months after treatment initiation and a PB patient came 6 times within the first 9 months after treatment initiation. Paediatric patients were defined as cases aged < 15 years.

Excel (Microsoft Excel for Mac, Version: 16.27 [19071500], 2019) and SPSS statistics (IBM, Version 29.0.1.0.) were used for data management, descriptive analysis as well as preparation of tables and figures. Maps were created using QGIS (Version: 3.32.3-Lima). Population numbers were taken from the Center for Health Information Management, 2022 [16].

## Results

### Baseline characteristics

Data for 823 cases were recorded in the NLCP registry during the observational period. Seven cases were not included in this analysis. Out of these, five were identified as relapses and two were excluded due to inconsistencies in the available age information. The total numbers of new leprosy cases in the final data set were 258 in 2017, 278 in 2018, and 280 in 2019. The mean age of patients at presentation across all three years was 43.7 years (SD ± 18.2). The diagnosis of leprosy was mainly based on clinical presentation. Microscopic diagnosis of slit skin smear was carried out in 25 out of 816 patients (3.1%). Out of those evaluated, 18 (72%) had a positive result for *Mycobacterium leprae*. Slit skin smears were exclusively used as diagnostic tool in patients clinically diagnosed with MB leprosy.

Detailed case numbers allocated to each region are displayed in Table 1 and S1 Fig.

**Table 1:** New leprosy cases reported to the NLCP between 2017 and 2019 by region.

Region	Reported number of cases (n)			Total/Region
	2017	2018	2019	
**Ashanti**	35	27	25	87
**Brong Ahafo**	29	29	34	92
**Central**	13	28	13	54
**Eastern**	39	40	36	115
**Greater-Accra**	9	12	19	40
**Northern**	14	17	23	54
**Upper East**	19	30	36	85
**Upper West**	35	39	52	126
**Volta**	34	33	25	92
**Western**	31	23	17	71
**Total Ghana**	258	278	280	816

**Fig 1:**
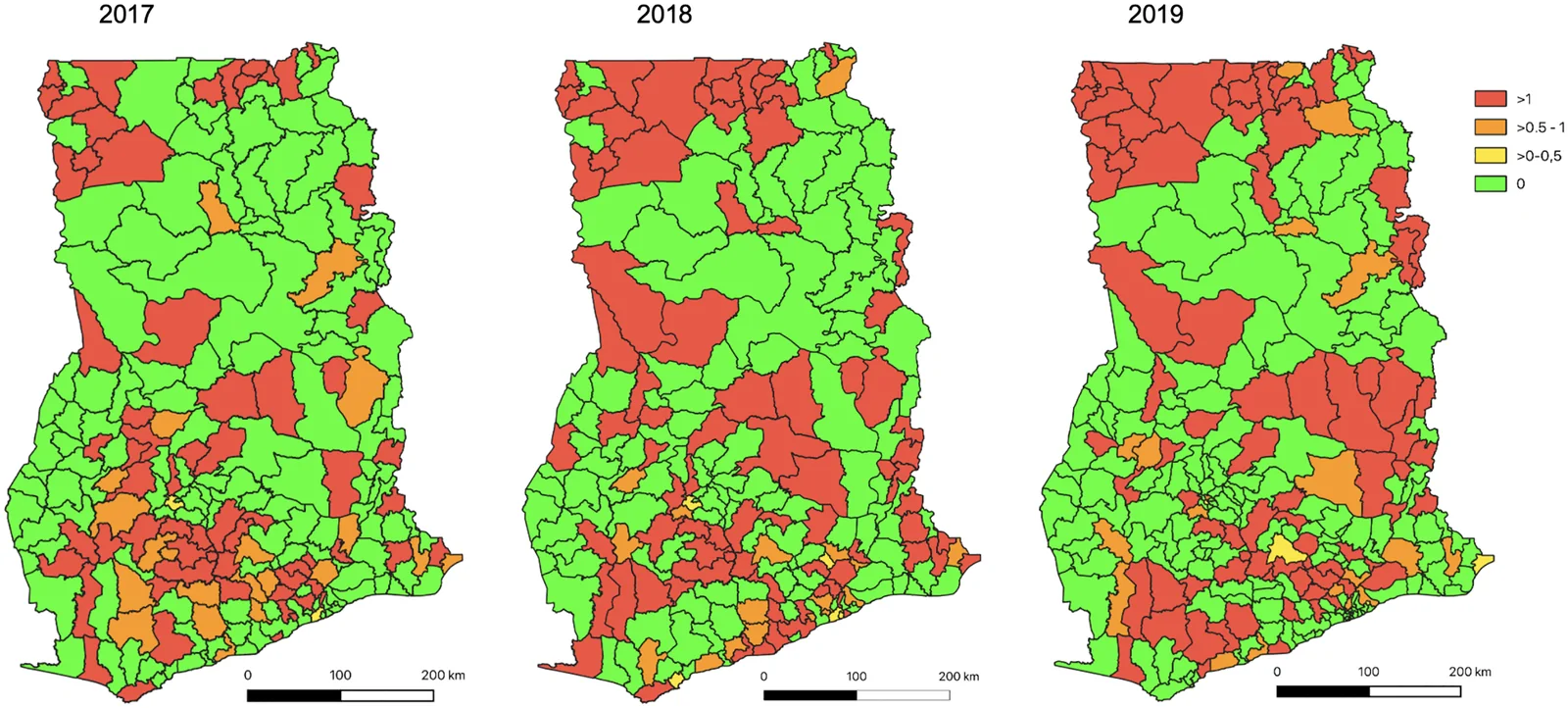
New leprosy case detection rate per 100,000 population per district in 2017, 2018 and 2019. (Shapefile source: 2017-2018: https://doi.org/10.5281/zenodo.21277794; 2019: https://www.geoboundaries.org/countryDownloads.html).

**Fig 2:**
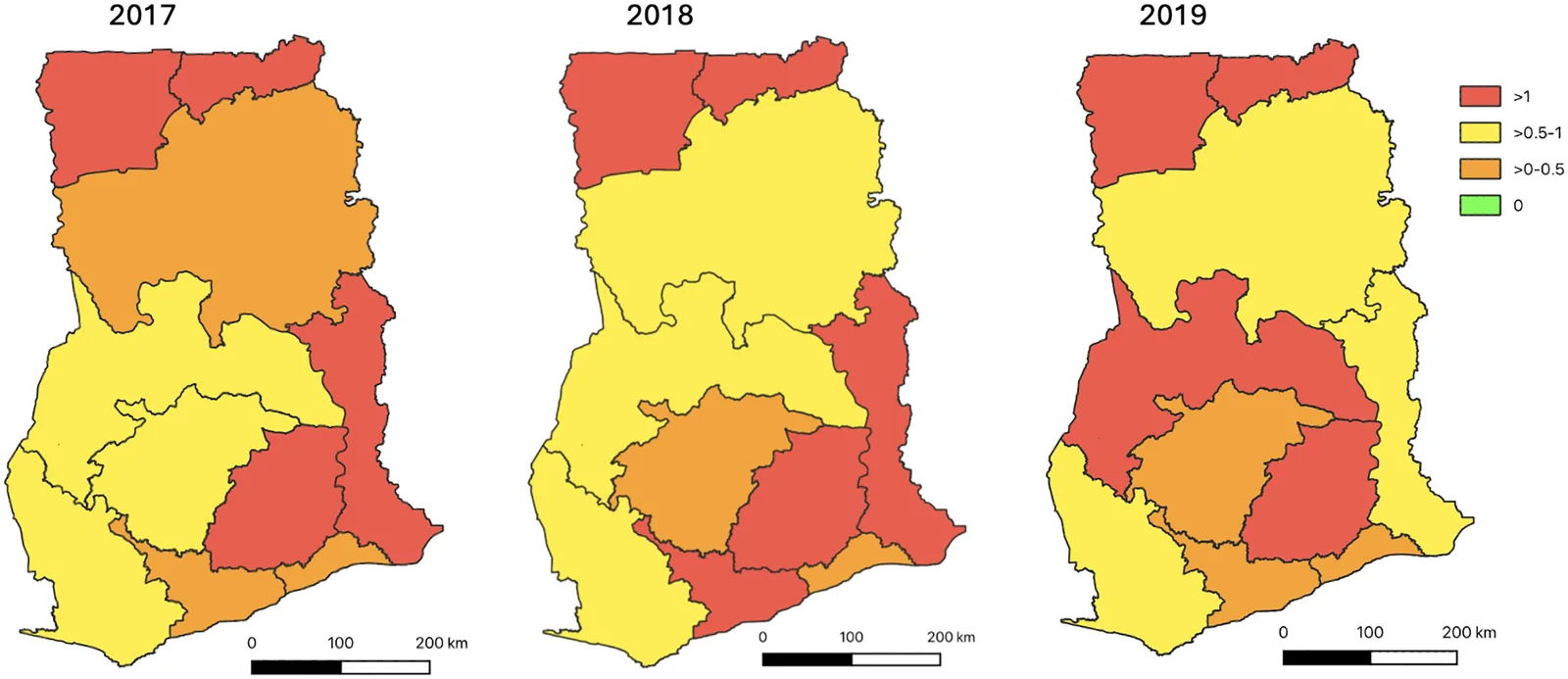
New leprosy case detection rate per 100,000 population per region in 2017, 2018 and 2019. (Shapefile source: 2017-2019: https://doi.org/10.5281/zenodo.21277794).

The Eastern and Upper West regions had the highest numbers of new cases were, while the Greater Accra, Central and Northern regions had the lowest case numbers. Across the three years (2017–2019), the mean case detection rate per 10,000 population in Ghana was 0.09 (0.09 in 2017, 0.09 in 2018, 0.09 in 2019). Fig 1 shows a detailed overview of the case detection rates per year in the respective districts, Fig 2. shows annual case detection rates in the regions.

### WHO programme performance indicators

The annual new case detection rates per 100,000 population for the whole country were 0.86 in 2017, 0.93 in 2018, and 0.90 in 2019. There were, however, regions with >1 case per 100,000 population (see Tables 2–4). Four percent of patients who were newly diagnosed with leprosy during the evaluated period were paediatric cases (n = 33). 43.9% of all new leprosy cases were female (n = 358).

**Table 2:** WHO core programmatic indicators per region for 2017.

Region	New cases/ 100,000 population	Age < 15 (%)	Female patients (%)	Grade II disability (%)	MB cases (%)	PB cases (%)	MB treatment completed (%)	PB treatment completed (%)
**Ashanti**	0.64	2.9	20.0	20.0	94.3	5.7	97.0	50.0
**Brong Ahafo**	0.93	3.4	58.6	37.9	89.7	10.3	100.0	100.0
**Central**	0.50	7.7	76.9	23.1	84.6	15.4	100.0	100.0
**Eastern**	1.19	12.8	30.6	0	100,0	0	100.0	0
**Greater-Accra**	0.19	0	44.4	0	100.0	0	100.0	0
**Northern**	0.49	0	42.9	0	100.0	0	100.0	0
**Upper East**	1.56	0	10.5	5.3	100.0	0	100.0	0
**Upper West**	4.32	2.9	60.0	0	91.4	8.6	90.6	100.0
**Volta**	1.36	0	47.1	0	100.0	0	100.0	0
**Western**	0.94	0	35.5	0	100.0	0	100.0	0
**Total**	0.86	3.5	41.1	8.5	96.1	3.9	98.4	90.0

**Table 3:** WHO core programmatic indicators per region for 2018.

Region 2018	New cases/ 100,000 population	Age < 15 (%)	Female patients (%)	Grade II disability (%)	MB cases (%)	PB cases (%)	MB treatment completed (%)	PB treatment completed (%)
**Ashanti**	0.49	7.4	37.0	33.3	100.0	0	100.0	0
**Brong Ahafo**	1.00	6.9	58.6	0	96.6	3.4	100.0	100.0
**Central**	1.11	0	46.4	7.1	96.4	3.6	92.6	100.0
**Eastern**	1.26	2.5	32.5	15.0	100.0	0	100.0	0
**Greater-Accra**	0.25	0	25.0	8.3	91.7	8.3	45.5	100.0
**Northern**	0.57	11.8	35.3	5.9	94.1	5.9	100.0	100.0
**Upper East**	2.41	0	53.3	6.7	93.3	6.7	100.0	100.0
**Upper West**	4.71	0	48.7	15.4	100.0	0	97.4	0
**Volta**	1.30	12.1	42.4	3.0	100.0	0	100.0	0
**Western**	0.69	0	39.1	0	100.0	0	100.0	0
**Total**	0.93	4.0	43.2	10.1	97.8	2.2	96.7	100.0

**Table 4:** WHO core programmatic indicators per region for 2019.

Region 2019	New cases/ 100,000 population	Age < 15 (%)	Female patients (%)	Grade II disability (%)	MB cases (%)	PB cases (%)
**Ashanti**	0.43	16.0	40.0	0	84.0	16.0
**Brong Ahafo**	1.16	2.9	50.0	2.9	91.2	8.8
**Central**	0.46	7.7	53.8	0	84.6	15.4
**Eastern**	1.04	2.8	38.9	5.6	100.0	0
**Greater-Accra**	0.37	5.3	26.3	10.5	94.7	5.3
**Northern**	0.76	0	39.1	4.3	100,0	0
**Upper East**	2.83	2.8	52.8	0	94.4	5.6
**Upper West**	6.12	0	55.8	3.8	96.2	3.8
**Volta**	0.96	8.0	48.0	16.0	100.0	0
**Western**	0.53	11.8	58.8	0	94.1	5.9
**Total**	0.90	4.6	47.1	4.3	94.6	5.4

At diagnosis, 657 patients (80.5%) presented with no disability, 97 (11.9%) with grade 1 disability (G1D), and 62 (7.6%) with a grade 2 disability. In the group of patients with no disability, 631 (96.0%) were adults and 26 (4.0%) were children. Among G1D patients, 91 (93.8%) were adults and 6 (6.2%) were children. While one child presented with G2D in 2019, the remaining G2D cases were adults. All patients presenting with G2D had underlying MB leprosy. The majority of G2D cases was male (n = 39, 62.9%).

Due to the long duration of a leprosy treatment course, treatment completion could only be evaluated for patients who were diagnosed with leprosy in 2017 and 2018. The treatment completion rate for PB cases was 100% in both years. The MB-case treatment completion rate was 98.4% in 2017 and 96.7% in 2018. Detailed percentages by indicator and by year are displayed in Tables 2–4.

### WHO programmatic 2020 targets

One paediatric case with G2D was detected within the three years of observation. The number of new leprosy cases with G2D per million population consistently remained below 1 each year, thus within the WHO target (see Table 5).

**Table 5:** Evaluable programmatic targets in Ghana per evaluated year.

Indicators	WHO target	Ghana 2017	Ghana 2018	Ghana 2019
1. Number of children diagnosed with leprosy and visible deformities (G2D) (n)	0	0	0	1
2. Rate of newly diagnosed leprosy patients with visible deformities (per million population)	< 1	0.74	0.94	0.39
Legislation allowing discrimination on basis of leprosy*	0	0	0	0

*No discriminating legislation in place in Ghana, information shared by GHS.

## Discussion

This manuscript presents a retrospective analysis of line list data collected by the Ghanaian NLCP between 2017 and 2019 including new leprosy patients that completed their treatment within this period. It was evaluated whether key data from Ghana met the performance criteria defined by the WHO in the Global Leprosy Strategy 2016–2020. A total of 816 new leprosy cases were evaluated. The annual new case detection per 100,000 population for Ghana remained below 1 case throughout the study period. Individual regions however, registered with >1 case per 100,000 population, indicating regional differences in disease burden and surveillance. The overall number of new leprosy cases afflicted with G2D remained <1 per million population in each of the three years, thereby fulfilling the requirements of the WHO. However, the goal of reducing paediatric leprosy cases with G2D to zero, was not met, with one child in 2019.

The paediatric population is a main focus of the WHO Global Leprosy Strategy 2016–2020 as it serves as indicator for the status of transmission and disease-related disability in a country. The highest proportion of paediatric cases among newly diagnosed leprosy cases in Ghana was observed in 2019 with 4.6% which was, however, lower than the global proportion of 7.4% presented for the same year [17]. Togo showed proportions of 7.4%, 4.0% and 6.3% in 2017, 2018 and 2019 respectively [18].

The proportion of leprosy patients with G2D among all newly detected cases is an indicator for leprosy-related awareness and education in a population and among health care workers. At the same time, it reflects the access to adequate healthcare, control of leprosy transmission and social barriers [12]. When new cases present with G2D at diagnosis, this can indicate a diagnostic delay. Out of the whole patient group investigated in this study, 62 (7.6%) cases presented with G2D at first diagnosis. This is considerably lower compared to the 22% shown by a meta-analysis of studies conducted in different parts of the world on the epidemiology of leprosy published between 2010 and 2020 but higher than the global proportion of 5.3% shown in the 2019 Global leprosy update [17,19]. Data from neighbouring Benin even showed 32.0% of G2D in new leprosy cases in 2018 [20]. The WHO target of less than 1 case was achieved by Ghana each year evaluated here [11].

Of particular interest in the evaluation of transmission and early case detection is the proportion of G2D cases among paediatric patients. A low rate of G2D in children indicates a more robust health care system with regards to early detection, management and control resulting in lower transmission. As suggested by WHO, the absence of G2D in children with leprosy serves as indicator of reduced disease transmission and a lower level of G2D in the whole population [11,12]. Unfortunately, the WHO target defined as the complete absence of G2D among children with leprosy could not be reached by Ghana in 2019 in Ahsanti. This indicates that there might still be crucial gaps in the local leprosy control strategy with hidden undiagnosed cases in the surrounding areas, as early-stage disease in the child remained undetected until it developed G2D associated with more visible signs of leprosy.

Throughout the observational period, more men than women were afflicted with leprosy, however with a tendency towards closure of the gender-gap (41.1% women in 2017, 43.2% in 2018 and 47.1% in 2019). Bakoubayi and colleagues demonstrated female proportions of 39.7%, 49.3% and 54% in 2017, 2018 and 2019, respectively, in Togo which actually represents a snapshot of fluctuating proportions of females around 50% over a 12-year period, with an overall slight increase [18]. Benin reported 45.9% and 38% in 2017 and 2018, respectively, which likewise represents a snapshot of fluctuating proportions of female cases around 50% over a period of 12 years, this time with an overall slight decrease [20]. Interestingly, these proportions are all well above the proportion of women diagnosed in the whole African Region (29.8%). This may be explained by the increased efforts in new case detection along with reduced barriers to healthcare for women in Ghana and the other countries that take the effort to analyse and publish their data in peer-reviewed scientific journals [17]. It is, however, noteworthy that huge regional disparities in the proportion of women were seen in this analysis, e.g., 17% in the Upper East versus 77% in the Central region in 2017. This requires further scrutiny and investigation.

In this dataset, the proportion of MB cases among all new leprosy cases was consistently above 94% and thereby considerably higher than the global average of 61% [12]. Benin reported 86.3% MB cases in 2017 and 90% in 2018 which was preceded by a gradual increase of MB cases over the previous years [20]. Togo reported 89.7, 92.0 an 88.9% of MB cases in 2017, 2018 and 2019 [18]. It seems that while regions with high prevalence detect more PB cases, regions with low prevalence detect more MB cases. PB leprosy patients show less severe symptoms than MB leprosy patients. This could therefore reflect the level of exposure of health care staff to leprosy and the resulting higher or lower level of experience in identifying leprosy signs and symptoms that is important for early diagnosis [20].

Besides a study by Simpson et al, this is the first systematic evaluation of programmatic data from the NLCP in Ghana focussing on leprosy and programme performance that is published in a peer-reviewed scientific journal [21]. This represents an important milestone. Limitations of this retrospective study are that the analysable data points were restricted to the data points available in the NLCP’s line list, therefore not all WHO performance milestones could be assessed. Furthermore, the NLCP depends on the collaboration of the reporting centres. In 2019, the NLCP reported major challenges in data collation such as delays and inaccuracies in monthly reports as well as loss of patient registers in some facilities which may account for incomplete and discordant data [14]. This was accompanied by a time-consuming verification and validation process. Generally, before the year 2020, summary surveillance data were monthly reported by the hospitals to the region and by the regions to the NLCP. These surveillance data were reported to the WHO. In a next step, the NLCP would go back to the hospitals to request more details on their individual leprosy cases and compile a line list with this information. This, however, would change the summary data previously reported to WHO. The line list would be more detailed and more accurate than the data from surveillance reporting. As a result, discrepant data from Ghana circulate from this period. This includes important discrepancies in G2D in newly diagnosed paediatric leprosy cases. At the same time this comes with the advantage that the data set presented in this analysis is the most accurate and complete one from the given period (cut-off date for line list updates: 17.01.2022).

In conclusion, this retrospective study shows that during 2017–2019, pre-Covid-19, Ghana partially fulfilled the requirements of the WHO as defined in the Global Leprosy Strategy 2016–2020. While the country performed comparatively well in many of the evaluated indicators, one paediatric case of G2D at first presentation was observed. To bring this number down to zero, extended active case finding strategies specifically tailored to the paediatric population are recommended. This could include integration of leprosy screening into child health services as well as school-based screening programmes throughout the country. Also, distinct regional deviations indicate that targeted interventions and surveillance by the NLCP should be extended to support early detection and disability prevention particularly in higher burden regions. The data presented in this report serve as a baseline for follow-up analyses with 2020–2025 leprosy data under the new WHO Global Leprosy Strategy as well as for evaluations of the leprosy situation in Ghana during and after the Covid-19 era [22,23]. It further serves as comparator for disease control programmes of other countries the region to inspire exchange and collaboration.

## Supporting information

S1 Fig Numbers of new leprosy cases per region in 2017, 2018 and 2019. (Shapefile source: 2017–2019: https://doi.org/10.5281/zenodo.21277794).
(TIF)

S1 Checklist (Source: https://www.strobe-statement.org/checklists/). (PDF)
