# Supplementary material for: Surveillance data and progress assessment of the targets of the WHO Global Leprosy Strategy 2016–2020: A retrospective registry-based analysis in Ghana
Source: PLoS Negl Trop Dis. 2026 Aug 10;20(8):e0014604. doi: 10.1371/journal.pntd.0014604 (PMC13502690; doi:10.1371/journal.pntd.0014604)
Supplement: Supplementary file 1 [file pntd.0014604.s002.pdf]

STROBE Statement—checklist of items that should be included in reports of observational studies

	Item No.	Recommendation	Page No.	Relevant text from manuscript
**Title and abstract**	1	(*a*) Indicate the study's design with a commonly used term in the title or the abstract	1	*Title:* "A retrospective registry-based analysis in Ghana"
		(*b*) Provide in the abstract an informative and balanced summary of what was done and what was found	2	Demographic as well as clinical data from the registry of the Ghanaian NLCP from 2017 to 2019 were reviewed and described. Evaluated targets and indicators for WHO programme monitoring included new case detection rates, proportion of new cases with G2D, biological sex, paediatric cases, proportions of multibacillary (MB) and paucibacillary (PB) cases and treatment completion rates. Data were further categorized according to region and year.<br>A total of 816 new leprosy cases were evaluated in this analysis. Of these, 358 (43.9%) were female cases and 33 (4. 0%) were paediatric cases. While the annual new case detection rate per 100,000 population in Ghana was 0.86 in 2017, 0.93 in 2018, and 0.90 in 2019, individual regions, particularly the Upper West Region, remained consistently above the threshold of 1 new case per 100,000 (4.32, 4.71, 6.12). Of all new cases, 7.6% (n=62) presented with G2D, the rate remained <1 per million population in each of the three years. One paediatric G2D case was reported in 2019. Annual treatment completion rates of MB and PB cases were well over 90% in 2017 and 2018.
**Introduction**				
Background/rationale	2	Explain the scientific background and rationale for the investigation being reported	4-5	The WHO's Global Leprosy Strategy 2016-2020 defined three main goals for the elimination of leprosy: reduction of cases with grade 2 disability (G2D) among paediatric leprosy patients to zero, reduction of new leprosy cases with G2D to less than one case per million population and reduction of countries with legislation allowing leprosy-associated discrimination to zero (11). In addition, other so-called "programme performance indicators" were defined to enable monitoring of progress (11, 12). Another aim of WHO's Global Leprosy Strategy 2016-2020 was to further eliminate leprosy at the subnational level by initiating action, ensuring accountability

				and promoting inclusivity, to set the course for reaching sustainable development goal 3 good health and wellbeing for all by 2030 (12).<br><br>While country-specific data are also reported to and evaluated by the WHO on a regular basis, different regions of Ghana face individual challenges related to leprosy. To be able to address and monitor these challenges in a more targeted way, this retrospective registry-based cross-sectional analysis aimed to evaluate the available core programme indicators and elimination goals of NLCP in relation to the WHO's Global Leprosy Strategy 2016-2020.
Objectives	3	State specific objectives, including any prespecified hypotheses	5	[…] To be able to address and monitor these challenges in a more targeted way, this retrospective registry-based cross-sectional analysis aimed to evaluate the available core programme indicators and elimination goals of NLCP in relation to the WHO's Global Leprosy Strategy 2016-2020. To allow for more detailed monitoring and more targeted public health interventions by the NLCP, this descriptive analysis was done not only at the national but also at the regional level.
**Methods**				
Study design	4	Present key elements of study design early in the paper	1, 2	*Title:* Surveillance data and progress assessment of the targets of the WHO Global Leprosy Strategy 2016-2020: A retrospective registry-based analysis in Ghana<br><br>*Abstract:* Demographic as well as clinical data from the registry of the Ghanaian NLCP from 2017 to 2019 were reviewed and described. Evaluated targets and indicators for WHO programme monitoring included new case detection rates, proportion of new cases with G2D, biological sex, paediatric cases, proportions of multibacillary (MB) and paucibacillary (PB) cases and treatment completion rates. Data were further categorized according to region and year.
Setting	5	Describe the setting, locations, and relevant dates, including periods of recruitment, exposure, follow-up, and data collection	6	The NLCP routinely collects leprosy-related data from health care facilities all over Ghana in a country-wide registry. Until 2020, surveillance data were reported monthly by health centres to the region and then to the NLCP. Based on these data, the NLCP went back to the health centres to request more details and compiled a line list, which served as basis for this analysis. All patients who were newly diagnosed with leprosy between 2017 and 2019 were included in this retrospective cross-sectional analysis of

				data from the NLCP-related registry. Patients with first leprosy detection in 2019 were, however, excluded from the calculation of treatment completion rates as for them treatment completion was to be reached only beyond the observational period. Relapse cases and cases that newly presented in 2016, were excluded as well as cases with discrepant information between age and year of birth. Another case was excluded due to discrepant information between treatment date and year. No age restrictions were applied. In 2019, six new regions and additional districts were established in Ghana (15). This descriptive data analysis considers the 10 former regions which are Ashanti, Brong Ahafo, Central, Eastern, Greater-Accra, Northern, Upper East, Upper West, Volta, and the Western Region.<br><br>Unique identifiers were allocated by NLCP staff before the dataset was shared for further cleaning and analysis. Variables extracted from the database were year of first detection, gender, year of birth/age, residence, disease classification, skin smear date and result, maximum disability grade at diagnosis, date of treatment initiation, and treatment attendance.
Participants	6	(*a*) *Cross-sectional study*—Give the eligibility criteria, and the sources and methods of selection of participants	6	All patients who were newly diagnosed with leprosy between 2017 and 2019 were included in this retrospective cross-sectional analysis of data from the NLCP-related registry. Patients with first leprosy detection in 2019 were, however, excluded from the calculation of treatment completion rates as for them treatment completion was to be reached only beyond the observational period. Relapse cases and cases that newly presented in 2016, were excluded as well as cases with discrepant information between age and year of birth. Another case was excluded due to discrepant information between treatment date and year. No age restrictions were applied.
		(*b*) *Cohort study*—For matched studies, give matching criteria and number of exposed and unexposed<br>*Case-control study*—For matched studies, give matching criteria and the number of controls per case		NA

Variables	7	Clearly define all outcomes, exposures, predictors, potential confounders, and effect modifiers. Give diagnostic criteria, if applicable	7	[…] the following core programmatic indicators were calculated: annual new case detection (total number of new leprosy cases and rate per 100,000 of the population), proportion of grade 2 disability (G2D) cases among new cases, proportion of paediatric cases among new cases, proportion of MB cases among new cases, proportion of female cases among new cases, treatment completion rate of MB cases, and treatment completion rate of PB cases. Treatment was regarded as completed if a MB patient came to collect the medication 12 times within the first 18 months after treatment initiation and a PB patient came 6 times within the first 9 months after treatment initiation. Paediatric patients were defined as cases aged < 15 years.
Data sources/ measurement	8*	For each variable of interest, give sources of data and details of methods of assessment (measurement). Describe comparability of assessment methods if there is more than one group	6,7	Variables extracted from the database were year of first detection, gender, year of birth/age, residence, disease classification, skin smear date and result, maximum disability grade at diagnosis, date of treatment initiation, and treatment attendance. In order to compare the data available from the registry with the performance milestones defined in Global Leprosy Strategy 2016-2020 the following core programmatic indicators were calculated: annual new case detection (total number of new leprosy cases and rate per 100,000 of the population), proportion of grade 2 disability (G2D) cases among new cases, proportion of paediatric cases among new cases, proportion of MB cases among new cases, proportion of female cases among new cases, treatment completion rate of MB cases, and treatment completion rate of PB cases. Treatment was regarded as completed if a MB patient came to collect the medication 12 times within the first 18 months after treatment initiation and a PB patient came 6 times within the first 9 months after treatment initiation. Paediatric patients were defined as cases aged < 15 years.
Bias	9	Describe any efforts to address potential sources of bias	6, 8, 17	Until 2020, surveillance data were reported monthly by health centres to the region and then to the NLCP. Based on these data, the NLCP went back to the health centres to request more details and compiled a line list, which served as basis for this analysis.<br><br>Relapse cases and cases that newly presented in 2016, were excluded as well as cases with discrepant information between age and year of birth.<br><br>Out of these, five were identified as relapses and two were excluded due to inconsistencies in the available age information.

				Limitations of this retrospective study are that the analysable data points were restricted to the data points available in the line list, therefore not all WHO performance milestones could be assessed. Furthermore, the NLCP depends on the collaboration of the reporting centres.
Study size	10	Explain how the study size was arrived at	6	All patients who were newly diagnosed with leprosy between 2017 and 2019 were included in this retrospective cross-sectional analysis of data from the NLCP-related registry. Patients with first leprosy detection in 2019 were, however, excluded from the calculation of treatment completion rates as for them treatment completion was to be reached only beyond the observational period. Relapse cases and cases that newly presented in 2016 were excluded, as well as cases with discrepant information between age and year of birth.

Continued on next page

Quantitative variables	11	Explain how quantitative variables were handled in the analyses. If applicable, describe which groupings were chosen and why	6	In order to compare the data available from the registry with the performance milestones defined in Global Leprosy Strategy 2016-2020 the following core programmatic indicators were calculated: annual new case detection (total number of new leprosy cases and rate per 100,000 of the population), proportion of grade 2 disability (G2D) cases among new cases, proportion of paediatric cases among new cases, proportion of MB cases among new cases, proportion of female cases among new cases, treatment completion rate of MB cases, and treatment completion rate of PB cases.
Statistical methods	12	(*a*) Describe all statistical methods, including those used to control for confounding	6-7	In order to compare the data available from the registry with the performance milestones defined in Global Leprosy Strategy 2016-2020 the following core programmatic indicators were calculated: annual new case detection (total number of new leprosy cases and rate per 100,000 of the population), proportion of grade 2 disability (G2D) cases among new cases, proportion of paediatric cases among new cases, proportion of MB cases among new cases, proportion of female cases among new cases, treatment completion rate of MB cases, and treatment completion rate of PB cases.<br><br>Excel (Microsoft Excel for Mac, Version: 16.27 [19071500], 2019) and SPSS statistics (IBM, Version 29.0.1.0.) were used for data management, descriptive analysis as well as preparation of tables and figures. Maps were created using QGIS (Version: 3.32.3-Lima).
		(*b*) Describe any methods used to examine subgroups and interactions		NA
		(*c*) Explain how missing data were addressed	6	There were no missing data in the dataset.<br><br>Until 2020, surveillance data were reported monthly by health centres to the region and then to the NLCP. Based on these data, the NLCP went back to the health centres to request more details and compiled a line list, which served as basis for this analysis.
		(*d*) *Cross-sectional study*—If applicable, describe analytical methods taking account of sampling strategy		NA
		(*e*) Describe any sensitivity analyses		NA
**Results**				

Participants	13*	(a) Report numbers of individuals at each stage of study—eg numbers potentially eligible, examined for eligibility, confirmed eligible, included in the study, completing follow-up, and analysed	8	Data for 823 cases were recorded in the NLCP registry during the observational period. Seven cases were not included in this analysis. Out of these, five were identified as relapses and two were excluded due to inconsistencies in the available age information. The total numbers of new leprosy cases in the final data set were 258 in 2017, 278 in 2018, and 280 in 2019.
		(b) Give reasons for non-participation at each stage	8	Out of these, five were identified as relapses and two were excluded due to inconsistencies in the available age information.
		(c) Consider use of a flow diagram		Not included as exclusion numbers are very easy to understand.
Descriptive data	14*	(a) Give characteristics of study participants (eg demographic, clinical, social) and information on exposures and potential confounders	8, 10, 14	The mean age of patients at presentation across all three years was 43.7 years (SD ±18.2). The diagnosis of leprosy was mainly based on clinical presentation. Microscopic diagnosis of slit skin smear was carried out in 25 out of 816 patients (3.1%). Out of those evaluated, 18 (72%) had a positive result for *Mycobacterium leprae*. Slit skin smears were exclusively used as diagnostic tool in patients clinically diagnosed with MB leprosy.<br><br>The annual new case detection rates per 100,000 population for the whole country were 0.86 in 2017, 0.93 in 2018, and 0.90 in 2019. There were, however, regions with >1 case per 100,000 population (see Tables 2-4). Four percent of patients who were newly diagnosed with leprosy during the evaluated period were paediatric cases (n = 33). 43.9% of all new leprosy cases were female (n = 358).<br>At diagnosis, 657 patients (80.5%) presented with no disability, 97 (11.9%) with grade 1 disability (G1D), and 62 (7.6%) with a grade 2 disability. In the group of patients with no disability, 631 (96.0%) were adults and 26 (4.0%) were children. Among G1D patients, 91 (93.8%) were adults and 6 (6.2 %) were children. While one child presented with G2D in 2019, the remaining G2D cases were adults. All patients presenting with G2D had underlying MB leprosy. The majority of G2D cases was male (n = 39, 62.9%).<br><br>One paediatric case with G2D was detected within the three years of observation. The number of new leprosy cases with G2D per million population consistently remained below 1 each year, thus within the WHO target (see Table 5).

		(b) Indicate number of participants with missing data for each variable of interest		There are no missing data in this dataset.
		(c) *Cohort study*—Summarise follow-up time (eg, average and total amount)		NA
Outcome data	15*	*Cohort study*—Report numbers of outcome events or summary measures over time		NA
		Case-control study—Report numbers in each exposure category, or summary measures of exposure		NA
		Cross-sectional study—Report numbers of outcome events or summary measures	10, 14	The annual new case detection rates per 100,000 population for the whole country were 0.86 in 2017, 0.93 in 2018, and 0.90 in 2019. There were, however, regions with >1 case per 100,000 population (see Tables 2-4). Four percent of patients who were newly diagnosed with leprosy during the evaluated period were paediatric cases (n = 33).<br>43.9% of all new leprosy cases were female (n = 358).<br>At diagnosis, 657 patients (80.5%) presented with no disability, 97 (11.9%) with grade 1 disability (G1D), and 62 (7.6%) with a grade 2 disability. In the group of patients with no disability, 631 (96.0%) were adults and 26 (4.0%) were children. Among G1D patients, 91 (93.8%) were adults and 6 (6.2 %) were children. While one child presented with G2D in 2019, the remaining G2D cases were adults. All patients presenting with G2D had underlying MB leprosy. The majority of G2D cases was male (n = 39, 62.9%).<br>Due to the long duration of a leprosy treatment course, treatment completion could only be diagnosed with leprosy in 2017 and 2018. The treatment completion rate for PB cases was treatment completion rate was 98.4% in 2017 and 96.7% in 2018. Detailed percentages by Tables 2-4.<br><br>One paediatric case with G2D was detected within the three years of observation. The number of new leprosy cases with G2D per million population consistently remained below 1 each year, thus within the WHO target (see Table 5).
Main results	16	(*a*) Give unadjusted estimates and, if applicable, confounder-adjusted estimates and their precision (eg, 95% confidence interval). Make clear which	8, 10, 14	The diagnosis of leprosy was mainly based on clinical presentation. Microscopic diagnosis of slit skin smear was carried out in 25 out of 816 patients (3.1%). Out of those evaluated, 18 (72%) had a positive result for *Mycobacterium leprae*.

	confounders were adjusted for and why they were included	The annual new case detection rates per 100,000 population for the whole country were 0.86 in 2017, 0.93 in 2018, and 0.90 in 2019. There were, however, regions with >1 case per 100,000 population (see Tables 2-4). Four percent of patients who were newly diagnosed with leprosy during the evaluated period were paediatric cases (n = 33). 43.9% of all new leprosy cases were female (n = 358).<br>At diagnosis, 657 patients (80.5%) presented with no disability, 97 (11.9%) with grade 1 disability (G1D), and 62 (7.6%) with a grade 2 disability. In the group of patients with no disability, 631 (96.0%) were adults and 26 (4.0%) were children. Among G1D patients, 91 (93.8%) were adults and 6 (6.2 %) were children. While one child presented with G2D in 2019, the remaining G2D cases were adults. All patients presenting with G2D had underlying MB leprosy. The majority of G2D cases was male (n = 39, 62.9%).<br>Due to the long duration of a leprosy treatment course, treatment completion could only be evaluated for patients who were diagnosed with leprosy in 2017 and 2018. The treatment completion rate for PB cases was 100% in both years. The MB-case treatment completion rate was 98.4% in 2017 and 96.7% in 2018. Detailed percentages by indicator and by year are displayed in Tables 2-4.<br><br>Table 5
	(*b*) Report category boundaries when continuous variables were categorized	NA
	(*c*) If relevant, consider translating estimates of relative risk into absolute risk for a meaningful time period	NA

Continued on next page

Other analyses	17	Report other analyses done—eg analyses of subgroups and interactions, and sensitivity analyses		NA
**Discussion**				
Key results	18	Summarise key results with reference to study objectives	15	This manuscript presents a retrospective analysis of line list data collected by the Ghanaian NLCP between 2017 and 2019 including new leprosy patients that completed their treatment within this period. It was evaluated whether key data from Ghana met the performance criteria defined by the WHO in the Global Leprosy Strategy 2016–2020. A total of 816 new leprosy cases were evaluated. The annual new case detection per 100,000 population for Ghana remained below 1 case throughout the study period. Individual regions however, registered with >1 case per 100,000 population, indicating regional differences in disease burden and surveillance. The overall number of new leprosy cases afflicted with G2D remained <1 per million population in each of the three years, thereby fulfilling the requirements of the WHO. However, the goal of reducing paediatric leprosy cases with G2D to zero, was not met, with one child in 2019.
Limitations	19	Discuss limitations of the study, taking into account sources of potential bias or imprecision. Discuss both direction and magnitude of any potential bias	17	Limitations of this retrospective study are that the analysable data points were restricted to the data points available in the NLCP's line list, therefore not all WHO performance milestones could be assessed. Furthermore, the NLCP depends on the collaboration of the reporting centres. In 2019, the NLCP reported major challenges in data collation such as delays and inaccuracies in monthly reports as well as loss of patient registers in some facilities which may account for incomplete and discordant data (14). This was accompanied by a time-consuming verification and validation process. Generally, before the year 2020, summary surveillance data were monthly reported by the hospitals to the region and by the regions to the NLCP. These surveillance data were reported to the WHO. In a next step, the NLCP would go back to the hospitals to request more details on their individual leprosy cases and compile a line list with this information. This, however, would change the summary data previously reported to WHO. The line list would be more detailed and more accurate than the data from surveillance reporting. As a result, discrepant data from Ghana

				circulate from this period. This includes important discrepancies in G2D in newly diagnosed paediatric leprosy cases. At the same time this comes with the advantage that the data set presented in this analysis is the most accurate and complete one from the given period (cut-off date for line list updates: 17.01.2022).
Interpretation	20	Give a cautious overall interpretation of results considering objectives, limitations, multiplicity of analyses, results from similar studies, and other relevant evidence	15-17	The proportion of leprosy patients with G2D among all newly detected cases is an indicator for leprosy-related awareness and education in a population and among health care workers. At the same time, it reflects the access to adequate healthcare, control of leprosy transmission and social barriers (12). When new cases present with G2D at diagnosis, this can indicate a diagnostic delay. Out of the whole patient group investigated in this study, 62 (7.6%) cases presented with G2D at first diagnosis. This is considerably lower compared to the 22% shown by a meta-analysis of studies conducted in different parts of the world on the epidemiology of leprosy published between 2010 and 2020 but higher than the global proportion of 5.3% shown in the 2019 Global leprosy update (17, 19). Data from neighbouring Benin even showed 32.0% of G2D in new leprosy cases in 2018 (20). The WHO target of less than 1 case was achieved by Ghana each year evaluated here (11).<br>Of particular interest in the evaluation of transmission and early case detection is the proportion of G2D cases among paediatric patients. A low rate of G2D in children indicates a more robust health care system with regards to early detection, management and control resulting in lower transmission. As suggested by WHO, the absence of G2D in children with leprosy serves as indicator of reduced disease transmission and a lower level of G2D in the whole population (11, 12). Unfortunately, the WHO target defined as the complete absence of G2D among children with leprosy could not be reached by Ghana in 2019 in Ahsanti. This indicates that there might still be crucial gaps in the local leprosy control strategy with hidden undiagnosed cases in the surrounding areas, as early-stage disease in the child remained undetected until it developed G2D associated with more visible signs of leprosy.<br>Throughout the observational period, more men than women were afflicted with leprosy, however with a tendency towards closure of the gender-gap (41.1%

women in 2017, 43.2% in 2018 and 47.1% in 2019). Bakoubayi and colleagues demonstrated female proportions of 39.7%, 49.3% and 54% in 2017, 2018 and 2019, respectively, in Togo which actually represents a snapshot of fluctuating proportions of females around 50% over a 12-year period, with an overall slight increase (18). Benin reported 45.9% and 38% in 2017 and 2018, respectively, which likewise represents a snapshot of fluctuating proportions of female cases around 50% over a period of 12 years, this time with an overall slight decrease (20). Interestingly, these proportions are all well above the proportion of women diagnosed in the whole African Region (29.8%). This may be explained by the increased efforts in new case detection along with reduced barriers to healthcare for women in Ghana and the other countries that take the effort to analyse and publish their data in peer-reviewed scientific journals (17). It is, however, noteworthy that huge regional disparities in the proportion of women were seen in this analysis, e.g. 17% in the Upper East versus 77% in the Central region in 2017. This requires further scrutiny and investigation.

In this dataset, the proportion of MB cases among all new leprosy cases was consistently above 94% and thereby considerably higher than the global average of 61% (12). Benin reported 86.3% MB cases in 2017 and 90% in 2018 which was preceded by a gradual increase of MB cases over the previous years (20). Togo reported 89.7, 92.0 an 88.9% of MB cases in 2017, 2018 and 2019 (18). It seems that while regions with high prevalence detect more PB cases, regions with low prevalence detect more MB cases. PB leprosy patients show less severe symptoms than MB leprosy patients. This could therefore reflect the level of exposure of health care staff to leprosy and the resulting higher or lower level of experience in identifying leprosy signs and symptoms that is important for early diagnosis (20).

Besides a study by Simpson et al, this is the first systematic evaluation of programmatic data from the NLCP in Ghana focussing on leprosy and programme performance that is published in a peer-reviewed scientific journal (21). This represents an important milestone. Limitations of this retrospective study are that the analysable data points were restricted to the data points available in the NLCP's line list, therefore not all WHO performance milestones

				could be assessed. Furthermore, the NLCP depends on the collaboration of the reporting centres. In 2019, the NLCP reported major challenges in data collation such as delays and inaccuracies in monthly reports as well as loss of patient registers in some facilities which may account for incomplete and discordant data (14). This was accompanied by a time-consuming verification and validation process. Generally, before the year 2020, summary surveillance data were monthly reported by the hospitals to the region and by the regions to the NLCP. These surveillance data were reported to the WHO. In a next step, the NLCP would go back to the hospitals to request more details on their individual leprosy cases and compile a line list with this information. This, however, would change the summary data previously reported to WHO. The line list would be more detailed and more accurate than the data from surveillance reporting. As a result, discrepant data from Ghana circulate from this period. This includes important discrepancies in G2D in newly diagnosed paediatric leprosy cases. At the same time this comes with the advantage that the data set presented in this analysis is the most accurate and complete one from the given period (cut-off date for line list updates: 17.01.2022).
Generalisability	21	Discuss the generalisability (external validity) of the study results	15-18	Out of the whole patient group investigated in this study, 62 (7.6%) cases presented with G2D at first diagnosis. This is considerably lower compared to the 22% shown by a meta-analysis of studies conducted in different parts of the world on the epidemiology of leprosy published between 2010 and 2020 but higher than the global proportion of 5.3% shown in the 2019 Global leprosy update (17, 19). Data from neighbouring Benin even showed 32.0% of G2D in new leprosy cases in 2018 (20). The WHO target of less than 1 case was achieved by Ghana each year evaluated here (11).<br><br>Throughout the observational period, more men than women were afflicted with leprosy, however with a tendency towards closure of the gender-gap (41.1% women in 2017, 43.2% in 2018 and 47.1% in 2019). Bakoubayi and colleagues demonstrated female proportions of 39.7%, 49.3% and 54% in 2017, 2018 and 2019, respectively, in Togo which actually represents a snapshot of fluctuating proportions of females around 50% over a 12-year period, with an overall slight

			increase (18). Benin reported 45.9% and 38% in 2017 and 2018, respectively, which likewise represents a snapshot of fluctuating proportions of female cases around 50% over a period of 12 years, this time with an overall slight decrease (20). Interestingly, these proportions are all well above the proportion of women diagnosed in the whole African Region (29.8%). This may be explained by the increased efforts in new case detection along with reduced barriers to healthcare for women in Ghana and the other countries that take the effort to analyse and publish their data in peer-reviewed scientific journals (17). It is, however, noteworthy that huge regional disparities in the proportion of women were seen in this analysis, e.g. 17% in the Upper East versus 77% in the Central region in 2017. This requires further scrutiny and investigation.<br><br>In this dataset, the proportion of MB cases among all new leprosy cases was consistently above 94% and thereby considerably higher than the global average of 61% (12). Benin reported 86.3% MB cases in 2017 and 90% in 2018 which was preceded by a gradual increase of MB cases over the previous years (20). Togo reported 89.7, 92.0 an 88.9% of MB cases in 2017, 2018 and 2019 (18).<br><br>The data presented in this report serve as a baseline for follow-up analyses with 2020-2025 leprosy data under the new WHO Global Leprosy Strategy as well as for evaluations of the leprosy situation in Ghana during and after the Covid-19 era (22, 23). It further serves as comparator for disease control programmes of other countries the region to inspire exchange and collaboration.
**Other information**			
Funding	22	Give the source of funding and the role of the funders for the present study and, if applicable, for the original study on which the present article is based	No funding

*Give information separately for cases and controls in case-control studies and, if applicable, for exposed and unexposed groups in cohort and cross-sectional studies.

Note: An Explanation and Elaboration article discusses each checklist item and gives methodological background and published examples of transparent reporting. The STROBE checklist is best used in conjunction with this article (freely available on the Web sites of PLoS Medicine at http://www.plosmedicine.org/, Annals of Internal Medicine at http://www.annals.org/, and Epidemiology at http://www.epidem.com/). Information on the STROBE Initiative is available at www.strobe-statement.org.
